# Supplementary material for: Prevalence and risk factors of disability and anxiety in a retrospective cohort of 432 survivors of Coronavirus Disease-2019 (Covid-19) from China
Source: PLoS One. 2020 Dec 17;15(12):e0243883. doi: 10.1371/journal.pone.0243883 (PMC7746260; doi:10.1371/journal.pone.0243883)
Supplement: S1 Appendix — (DOCX) [file pone.0243883.s001.docx]

**S1 Appendix: Estimates of Risk Ratios and 95 % confidence intervals from log-linear Poisson regression with robust standard errors**

**Table S1. Outcome: One or more IADL problems (Lawton IADL scale, at least one item endorsed)^&^**

|  | *Unadjusted* | | *Adjusted* | |
| --- | --- | --- | --- | --- |
|  | RR (95% CI) | P | RR (95% CI) | P |
| Gender (reference: female) |  |  |  |  |
| male | 0.909 (0.709-1.163) | 0.447 | 0.985 (0.803-1.207) | 0.880 |
| Age group (reference: < 50 years) |  |  |  |  |
| 50-60 years | 1.806 (1.209-2.699) | 0.004 | 1.363 (0.966-1.922) | 0.078 |
| > 60 years | 4.699 (3.471-6.361) | < 0.001 | 2.518 (1.800-3.525) | < 0.001 |
| Province (reference: other province) |  |  |  |  |
| Hubei | 3.708 (2.800-4.911) | < 0.001 | 2.582 (1.810-3.684) | < 0.001 |
| Ethnicity (reference: Han) |  |  |  |  |
| Tibetan | 0.719 (0.485-1.068) | 0.102 | 2.391 (1.513-3.780) | < 0.001 |
| Current smoking (reference: no) |  |  |  |  |
| smoking, yes | 0.576 (0.357-0.930) | 0.024 | 0.823 (0.547-1.239) | 0.350 |
| Comorbid conditions (reference: none) |  |  |  |  |
| one | 2.211 (1.694-2.886) | < 0.001 | 1.101 (0.865-1.401) | 0.436 |
| multi | 2.436 (1.847-3.212) | < 0.001 | 1.143 (0.854-1.529) | 0.369 |
| Disease severity (reference: non-severe) |  |  |  |  |
| severe | 3.989 (3.060-5.201) | < 0.001 | 2.476 (1.801-3.404) | < 0.001 |
| Symptoms at admission (reference (<=1) |  |  |  |  |
| >1 | 1.602 (1.217-2.110) | 0.001 | 0.853 (0.656-1.109) | 0.234 |
| Scope of pneumonia at admission (reference: unilateral) |  |  |  |  |
| bilateral | 2.172 (1.308-3.605) | 0.003 | 1.446 (0.924-2.261) | 0.106 |

^&^ n= 431 for adjusted model and unadjusted model for age, n= 432 for all other models. RR = Risk Ratio. CI = Confidence Interval.

**Table S2. Outcome: At least moderate ADL dependence (Barthel Index, < 75)^&^**

|  | *Unadjusted* | | *Adjusted* | |
| --- | --- | --- | --- | --- |
|  | RR (95% CI) | P | RR (95% CI) | P |
| Gender (reference: female) |  |  |  |  |
|  | 1.121 (0.731-1.721) | 0.600 | 1.190 (0.810-1.747) | 0.376 |
| Age group (reference: < 50 years) |  |  |  |  |
| 50-60 years | 2.243 (0.938-5.362) | 0.069 | 1.652 (0.707-3.862) | 0.247 |
| > 60 years | 12.000 (6.145-23.419) | < 0.001 | 6.783 (3.155-14.586) | < 0.001 |
| Province (reference: other provinces) |  |  |  |  |
| Hubei | 3.048 (1.938-4.792) | < 0.001 | 1.441 (0.893-2.326) | 0.135 |
| Ethnicity (reference: Han) |  |  |  |  |
| Tibetan | 0.547 (0.261-1.145) | 0.109 | 1.749 (0.817-3.741) | 0.150 |
| Current smoking (reference: no) |  |  |  |  |
| smoking, yes | 0.866 (0.454-1.653) | 0.663 | 1.125 (0.646-1.959) | 0.677 |
| Comorbid conditions (reference: none) |  |  |  |  |
| one | 3.505 (2.133-5.762) | < 0.001 | 1.344 (0.837-2.158) | 0.222 |
| multi | 4.572 (2.784-7.507) | < 0.001 | 1.344 (0.790-2.285) | 0.275 |
| Disease severity (reference: non-severe) |  |  |  |  |
| severe | 5.709 (3.473-9.382) | < 0.001 | 2.074 (1.146-3.755) | 0.016 |
| Symptoms at admission (reference (<=1) |  |  |  |  |
| >1 | 3.613 (2.000-6.527) | < 0.001 | 2.074 (1.129-3.810) | 0.019 |
| Scope of pneumonia at admission (reference: unilateral) |  |  |  |  |
| Bilateral | 2.095 (0.944-4.649) | 0.069 | 1.019 (0.489-2.120) | 0.961 |

^&^n= 431 for adjusted model and unadjusted model for age, n= 432 for all other models. RR = Risk Ratio. CI = Confidence Interval.

**Table S3. Outcome: Anxiety (Zung's self-reported anxiety scale, raw score ≥ 40)^&^**

|  | *Unadjusted* | | *Adjusted* | |
| --- | --- | --- | --- | --- |
|  | RR (95% CI) | P | RR (95% CI) | P |
| Gender (reference: female) |  |  |  |  |
| male | 1.014 (0.752-1.365) | 0.929 | 1.019 (0.790-1.315) | 0.883 |
| Age group (reference: < 50 years) |  |  |  |  |
| 50-60 years | 1.502 (1.011-2.231) | 0.004 | 1.087 (0.764-1.548) | 0.642 |
| > 60 years | 2.318 (1.650-3.255) | <0.001 | 1.037 (0.750-1.432) | 0.827 |
| Province (reference: other province) |  |  |  |  |
| Hubei | 3.804 (2.717-5.326) | <0.001 | 2.055 (1.422-2.972) | <0.001 |
| Ethnicity (reference: Han) |  |  |  |  |
| Tibetan | 0.082 (0.021-0.324) | <0.001 | 0.214 (0.050-0.916) | 0.038 |
| Current smoking (reference: no) |  |  |  |  |
| smoking, yes | 0.821 (0.514-1.312) | 0.410 | 0.973 (0.595-1.590) | 0.912 |
| Comorbid conditions (reference: none) |  |  |  |  |
| one | 1.899 (1.345-2.681) | <0.001 | 1.230 (0.920-1.644) | 0.163 |
| multi | 2.476 (1.772-3.462) | <0.001 | 1.206 (0.866-1.678) | 0.267 |
| Disease severity (reference: non-severe) |  |  |  |  |
| severe | 4.388 (3.167-6.080) | <0.001 | 2.533 (1.693-3.788) | <0.001 |
| Symptoms at admission (reference (<=1) |  |  |  |  |
| >1 | 2.303 (1.600-3.315) | <0.001 | 1.083 (0.745-1.574) | 0.677 |
| Scope of pneumonia at admission (reference: unilateral) |  |  |  |  |
| bilateral | 3.232 (1.574-6.635) | 0.001 | 1.726 (0.803-3.709) | 0.162 |
| Length of hospital stay (reference: <= 14 days) |  |  |  |  |
| > 14 days | 2.446 (1.633-3.664) | <0.001 | 1.482 (0.998-2.200) | 0.051 |

^&^n=407 for adjusted model and n=407 for unadjusted model for length of stay, n= 431 for unadjusted model for age, n= 432 for all other models. RR = Risk Ratio. CI = Confidence Interval.
