# Supplementary material for: Prevalence and risk factors of disability and anxiety in a retrospective cohort of 432 survivors of Coronavirus Disease-2019 (Covid-19) from China
Source: PLoS One. 2020 Dec 17;15(12):e0243883. doi: 10.1371/journal.pone.0243883 (PMC7746260; doi:10.1371/journal.pone.0243883)
Supplement: Attachment — Submitted filename: Response to Reviewers_20201010_SY_JR.docx [file pone.0243883.s003.docx]

**Manuscript PONE-D-20-25960**

**Response to Reviewers**

Dear Prof. Federici,

Thank you for giving us the opportunity to resubmit a revised draft of the manuscript “Prevalence and risk factors of disability and anxiety in a retrospective cohort of 432 survivors of Coronavirus Disease-2019 (Covid-19) from China” for publication in *PLoS One*. We appreciate the time and effort that you and the reviewers dedicated to providing feedback on our manuscript and are grateful for the insightful comments which have lead to valuable improvements of our paper. We have incorporated most of the suggestions made by the reviewers. Those changes are highlighted within the manuscript. Please see a point-by-point response to the reviewers’ comments and concerns below, with our response given in blue. All page and line numbers refer to the revised manuscript file with tracked changes.

Sincerely,

Jan Reinhardt (for the author team)

**Additional editorial requirements to the authors:**

1. Please provide additional details regarding participant consent. In the ethics statement in the Methods and online submission information, please ensure that you have specified what type you obtained (for instance, written or verbal, and if verbal, how it was documented and witnessed). If your study included minors, state whether you obtained consent from parents or guardians. If the need for consent was waived by the ethics committee, please include this information.

**Author response**: The informed consent process is now explained in more detail in the manuscript (lines 118-127, page 6):

‘All participating health professionals were instructed on implementation of the study protocol, informed consent procedures, and possible sources of bias by videoconference. Patients were informed by the health professionals either face to face (if they were still hospitalized) or via telephone or WeChat call (if they had already been discharged) about the study purpose and content, and that participation was voluntary and could be withdrawn at any time. Informed consent was then obtained either in written form or verbally. Verbal informed consent was obtained if the patients were either illiterate or had already been discharged. Verbally provided informed consent was witnessed by a second health professional and recorded electronically.’

2. We note that you have indicated that data from this study are available upon request. PLOS only allows data to be available upon request if there are legal or ethical restrictions on sharing data publicly.

**Author response**: The data that support the findings of this study have been made openly available via Mendeley Data at <http://dx.doi.org/10.17632/gwpnj5hph3.2> (Zhu, Siyi; Reinhardt, Jan D.; He, Chengqi (2020), “A retrospective cohort of 432 survivors of Coronavirus Disease-2019 (Covid-19) from China”, Mendeley Data, V2, doi: 10.17632/gwpnj5hph3.2).

**Reviewers' Comments to the Authors:**

**Reviewer 1**

Overall, I think that this is a useful study and there have been few publications to date that have considered the post-acute aspects of COVID-19. I do feel that the study itself has several limitations, however, which are largely highlighted by the authors.

**Author response**: Thank you.

1. My main issue is the lack of data regarding functional status prior to hospitalisation. The authors have used co-morbidity as a surrogate marker for this but given that hypertension is included as such, I do not think that there would necessarily be a strong correlation with functional dependence so I am not sure how valid it really is to use this. I would presume that the majority of patients admitted to hospital would have some background information gathered on their level of dependency/frailty as it usually forms a standard part of the history taking. However, given that this was a retrospective study and many of the patients were from Hubei province and admitted at the height of the pandemic, these details may not have been recorded and I also recognise that an incomplete data set may not add a huge amount to the interpretation of the findings.

**Author response:** The reviewer is correct that this is a limitation which however usually occurs with many health status measures unless people are participants in some large prospective cohort study that routinely gathers data on function before they develop a particular disease. The reviewer is also correct that comorbidity not necessarily correlates strongly with function in every case as other factors such as age and environmental factors may play a role. In the situation within which the patients were admitted However, comorbidity is the best proxy that is available with the current data. As noted by the reviewer, this imitation is discussed by us.

3. It is also interesting that a single recorded respiratory rate of above 30 is considered in isolation to be a marker of severe disease given that there could be many factors that could contribute to this (co-existent COPD or other chronic lung/heart condition/metabolic derangement/inaccurate documentation).

**Author response:** This is certainly correct. As discussed in the manuscript the criteria for classification of community acquired pneumonia as severe recommended by the American Thoracic Society are more strict. The criteria applied in the present data were the criteria recommended by the Chinese Health Commission at the time of the study. These criteria were applied in Chinese hospitals and they were thus relevant for clinical decision making. Given that little was known about the course of Covid-19 at the time of the study a more liberal definition of severe disease makes sense in order to alert clinicians early.

4. Although self-reported questionnaires may be less accurate than those recorded by the investigators, given that the findings are highly predictable, I suspect that overall would make little difference to the outcomes. I think that the study would be greatly enhanced by reassessing the same cohort at 3 months and it could be argued that this will provide more meaningful information regarding the longer term needs of those who have had COVID-19. However, given that early rehab/intervention programmes have long been established as key for effective rehabilitation, this study highlights the burden of disability at discharge and thus makes an important point regarding resource allocation and need for investment in post-acute COVID services (which in my experience has been somewhat overlooked.

**Author response:** We agree with the reviewer’s opinion. A protocol and grant proposal for an extended prospective cohort study following these and additional patients has been submitted to the Sichuan Bureau of Science and Technology and is currently under review. Another longitudinal study lead by one of the authors (JR) and funded by the Xi’an Bureau of Technology (XA2020-HWYZ-0043 ) and Xi’an Xidian Hospital on functional and psychological health status of Covid-19 survivors is currently ongoing. Findings will be published after conclusion of these studies which are intended to run over a 12 months period. We find it nevertheless meaningful to report data on functional disability and anxiety at discharge because this highlights two important issues also mentioned by the reviewer: 1) it could be beneficial to integrate early rehabilitation interventions into acute care, 2) many Covid-19 survivors are in need of additional medical services following discharge acute care.

5. There are a few typos (line 152 appears to be missing a word) and the introduction is slightly ‘media-like’ but overall I think it is reasonably well written and gets the points across well, in particular the limitations.

**Author response:** Thank you for pointing this out. We have thoroughly proofread the manuscript and reworded the sentence on page 7. The introduction is a little ‘media-like’ on purpose: We want to make sure to get the point across that we need health systems to plan for the rehabilitation of Covid-19 survivors.

**Reviewer 2**

This MS presents findings that make a strong case that those with more severe disease have increased disability and anxiety at discharge from the hospital.

**Author response:** Many thanks!

1. There are several areas of concern that the authors need to address before a final decision can be made regarding publication. While the results in the tables and Figures convey findings that are clearly statistically significant – e.g., Risk Ratios and 95%CIs for effects of Disease Severity, Ethnicity and Province on Anxiety in Figure 2 that do not overlap with 1 – it would help the reader appreciate these effects if the authors included P-values in the tables and text – e.g., “Having severe Covid-19 was the strongest risk factor for probable clinically relevant anxiety (P<0.00xx).” It would also be helpful to provide P-values for the age x disease severity effects reported in Table 2 and illustrated in Figure 3.

**Author response:** P-values have been added to the tables and effect sizes and confidence intervals or p-values have been integrated into the main text. P-values (if exact values are given) and confidence intervals are essentially equivalent and can be transformed into each other given the unstandardized effect size (see: Altman DG, Bland M. How to obtain the P value from a confidence interval. BMJ 2011, 343: d2304 and Altman DG, Bland M. How to obtain a confidence interval from a P value. BMJ 2011, 342: d2090). In the manuscript p-values are now provided for group comparison (severe vs non severe disease) with chi-squared test, Mann-Whitney U test, or logistic regression with regard to demographics, clinical characteristics and prevalence of outcomes. Risk Ratios and Incidence Rate Ratios with 95% confidence intervals are now provided in the manuscript text as well for the models. P-values have been added to all tables.

2. How are the three outcomes of IADL, ADL dependence and Anxiety correlated with each other? It is likely that those with more IADL and/or ADL dependence with have higher Anxiety levels, and it will be good to document and report whether that is the case. There is evidence in the literature that these factors are correlated (e.g., Clancy F, O'Connor DB, Prestwich A. Do Worry and Brooding Predict Health Behaviors? A Daily Diary Investigation. Int J Behav Med. 2020 Oct;27(5):591-601).

**Author response:** Information on the association between outcomes is now provided as detailed below.

Methods (Lines 192-195, page 9):

The strength of association between outcomes is reported as Cramer’s V with p-values from chi-squared tests. Cramer’s V ranges from zero (no association) to one (perfect association) with values from 0.3 to 0.5 indicating moderate effects and values above 0.5 indicating large effects.

Results (Lines 240-245, 15):

***Associations between outcomes***

Outcomes showed moderate to strong associations with Cramer’s V being 0.56 for the association between one or more IADL limitation and at least moderate dependence, 0.39 for the association between one or more IADL limitation and probable clinically relevant anxiety, and 0.33 for the association between at least moderate dependence and anxiety (all p < 0.001).

Discussion (Lines 362-364, page 21)

We found moderate to strong associations between outcomes, alluding to possible processes of mediation, i.e. indirect effects on one outcome triggered by the other outcomes.

3. . Given that the three outcomes are likely to be correlated, it could also be informative if the authors performed additional analyses to determine whether, e.g., the effect of disease severity to increase IADL is mediated by the effect of disease severity to increase Anxiety (or vice versa). This could be accomplished by using structural equation modeling to determine whether there is a significant indirect path – i.e., increased disease severity  increased Anxiety  increased IADL.

**Author response:** Thanks for this well taken comment. Mediation and possible indirect effects are now discussed. Given the cross-sectional nature of outcomes assessment and the novel and explorative character of this study, we however refrained from conducting complex analyses such as those involving SEM. Furthermore, such hypotheses had not been pre-specified. We discuss the issue under limitations.

Study Limitations (Lines 447-451, page 25)

Fifth, outcomes were correlated with each other pointing at possible mediation of effects. Indirect effects due to mediation were however not formally examined here given the cross-sectional nature of outcome assessment. Furthermore, we refrained from more complex analyses due to the novel character of the presented data and the therefore exploratory character of analysis.

4. In addition to Anxiety it would have also been informative if the authors had included a measure of depression – e.g., Zung Self-Rating Depression scale – to determine whether disease severity was also associated with the psychological disorder. They cited evidence (ref 31) showing that depression levels are increased in Chinese health Care Workers exposed to coronavirus disease, so it is likely that depression levels are also elevated in the patients with severe disease in the current study.

**Author response:** Thank you for this useful suggestion. We will incorporate this suggestion into the protocol of our upcoming prospective cohort study aimed at long-term follow up on functional and psychological status of Covid-19 survivors.

5. The authors note the important implication of their findings that early psychological/rehabilitation interventions could be useful in improving mental and physical health in Covid-19 survivors. There is evidence that training in cognitive behavioral stress management skills has been effective in reducing depression and anxiety in male cardiac surgery patients in Singapore (Bishop GD, Kaur D, Tan VL, Chua YL, Liew SM, Mak KH. Effects of a psychosocial skills training workshop on psychophysiological and psychosocial risk in patients undergoing coronary artery bypass grafting. Am Heart J. 2005 Sep;150(3):602-9) and Chinese medical students (Li C, Chu F, Wang H, Wang XP. Efficacy of Williams LifeSkills training for improving psychological health: a pilot comparison study of Chinese medical students. Asia Pac Psychiatry. 2014 Jun;6(2):161-9).

**Author response:** Thank you. We have reviewed the relevant evidence and cited the respective references in the revised manuscript.
